# Processing by rhomboid protease is required for *Providencia stuartii* TatA to interact with TatC and to form functional homo-oligomeric complexes

**DOI:** 10.1111/j.1365-2958.2012.08080.x

**Published:** 2012-05-17

**Authors:** Maximilian J Fritsch, Martin Krehenbrink, Michael J Tarry, Ben C Berks, Tracy Palmer

**Affiliations:** 1 Division of Molecular Microbiology, College of Life Sciences, University of DundeeDundee DD1 5EH, UK; 2 Department of Biochemistry, University of OxfordSouth Parks Road, Oxford OX1 3QU, UK

## Abstract

The twin arginine transport (Tat) system transports folded proteins across the prokaryotic cytoplasmic membrane and the plant thylakoid membrane. In *Escherichia coli* three membrane proteins, TatA, TatB and TatC, are essential components of the machinery. TatA from *Providencia stuartii* is homologous to *E. coli* TatA but is synthesized as an inactive pre-protein with an N-terminal extension of eight amino acids. Removal of this extension by the rhomboid protease AarA is required to activate *P. stuartii* TatA. Here we show that *P. stuartii* TatA can functionally substitute for *E. coli* TatA provided that the *E. coli* homologue of AarA, GlpG, is present. The oligomerization state of the *P. stuartii* TatA pro-protein was compared with that of the proteolytically activated protein and with *E. coli* TatA. The pro-protein still formed small homo-oligomers but cannot form large TatBC-dependent assemblies. In the absence of TatB, *E. coli* TatA or the processed form of *P. stuartii* TatA form a complex with TatC. However, this complex is not observed with the pro-form of *P. stuartii* TatA. Taken together our results suggest that the *P. stuartii* TatA pro-protein is inactive because it is unable to interact with TatC and cannot form the large TatA complexes required for transport.

## Introduction

Protein transport across the cytoplasmic membrane of prokaryotes and the thylakoid membrane of plant chloroplasts proceeds by one of two general mechanisms. The Sec pathway uses the energy of ATP hydrolysis and the transmembrane proton electrochemical gradient (Δ*p*) to drive the transport of unfolded proteins across the membrane (Driessen and Nouwen, 2008). By contrast, the Tat pathway transports folded proteins, in a reaction powered solely by Δ*p* (Cline and Theg, 2007; Palmer *et al*., 2010b). Proteins are directed to each of these pathways by the presence of cleavable N-terminal signal peptides. The signal peptides of Tat substrates, unlike Sec signal peptides, contain a highly conserved twin arginine motif which is essential for efficient targeting of substrates to the Tat transport machinery (Berks, 1996; Stanley *et al*., 2000).

In Gram-negative bacteria, such as *Escherichia coli*, the Tat machinery is made up of three membrane-bound proteins, TatA, TatB and TatC (Bogsch *et al*., 1998; Sargent *et al*., 1998; 1999; Weiner *et al*., 1998). In *E. coli* a fourth protein, TatE, is a functional homologue of TatA and forms a minor component of the Tat machinery (Sargent *et al*., 1998; Jack *et al*., 2001). In *E. coli* and closely related bacteria TatD is encoded by the fourth gene in the *tatABCD* operon, but is a cytoplasmic nuclease with no role in Tat transport (Wexler *et al*., 2000; Lindenstrauss *et al*., 2010). The three major Tat components form two different types of multimeric complexes in *E. coli* membranes. A complex of TatB and TatC can be isolated which contains each protein in a 1:1 molar ratio (Bolhuis *et al*., 2001). The exact number of TatB and TatC proteins within this complex is unknown, but it is probably between six and eight of each subunit (Bolhuis *et al*., 2001; Richter and Bruser, 2005; Tarry *et al*., 2009). Low levels of TatA co-purify with the TatBC complex when all three proteins are overproduced, but the complex lacks TatA when purified from cells expressing *tatABC* at native levels (McDevitt *et al*., 2005; 2006). The TatBC complex functions as a receptor for Tat substrates, with the twin arginine motif of the substrate signal peptide being recognized by TatC (Cline and Mori, 2001; de Leeuw *et al*.*,* 2002; Alami *et al*., 2003; Tarry *et al*., 2009).

The TatA protein comprises a single transmembrane helix, followed by an amphipathic helix and an unstructured C-terminal tail (Porcelli *et al*., 2002; Lee *et al*., 2006). The N-terminus of TatA is located at the periplasmic side of the membrane (Lee *et al*., 2006; Koch *et al*., 2012). Purified TatA forms a series of large homo-oligomers. Analysis of these TatA complexes by negative stain electron microscopy reveals ring-shaped structures with a range of different diameters in which a large central cavity is enclosed at one end (Gohlke *et al*., 2005). The variation in diameter results from differences in the number of TatA subunits while the presence of an enclosed cavity suggests that TatA complexes form transport channels of different internal size (Gohlke *et al*., 2005). Large assemblies of TatA have also been seen *in vivo* when a C-terminally YFP-tagged variant of TatA was produced at native levels (Leake *et al*., 2008). However, in the absence of TatB or TatC, the large assemblies of YFP-tagged TatA were not seen. Instead, fluorescence recovery after photobleaching (FRAP) experiments are consistent with TatA–YFP being arranged as small, possibly tetrameric units (Leake *et al*., 2008). Cross-linking studies of the chloroplast TatA orthologue Tha4 have also been interpreted as showing this protein in a tetramer state in resting thylakoid membranes (Dabney-Smith and Cline, 2009).

The requirement for TatBC in the assembly of large TatA–YFP oligomers indicates at least transient interactions between TatA with TatBC. This inference is supported by cross-linking experiments in thylakoids which detected Tha4 interactions with the thylakoid equivalent of the TatBC complex in the presence of substrate and a Δ*p* (Mori and Cline, 2002). These cross-links were no longer observed once the substrate had passed across the membrane indicating that TatA-TatBC interactions are transitory and occur only during active protein transport (Mori and Cline, 2002). Nevertheless, a recent cross-linking study suggests that monomeric TatA may associate with TatBC even in the absence of substrate. Contacts were observed between the TatA transmembrane helix and TatC, and between the TatA amphipathic helix and TatB (Frobel *et al*., 2011).

The Tat machineries of some Gram-positive bacteria and of archaea are comprised only of TatA and TatC proteins in contrast to the three-component TatB-containing systems found in Gram-negative bacteria and plant chloroplasts (Jongbloed *et al*., 2004; Dilks *et al*., 2005). However, protein purification studies indicate that the two-component Tat system of the Gram-positive bacterium *Bacillus subtilis* still forms two distinct membrane-bound complexes, one of which contains TatA and TatC proteins, and the second of which contains only TatA (Barnett *et al*., 2008; 2009). The physiological relevance of a large cytoplasmic aggregate of *B. subtilis* TatA protein that is seen in addition to membrane-bound TatA in the native organism, and also upon heterologous production in *E. coli,* is not clear (Westermann *et al*., 2006; Barnett *et al*., 2009). It is likely that the two- and three-component Tat machineries operate by a similar mechanism despite their differences in subunit composition. This hypothesis is supported by the isolation of point mutations in *tatA* that permit function of the *E. coli* Tat machinery in the absence of TatB (Blaudeck *et al*., 2005).

Recently it was demonstrated that the TatA protein from the enterobacterium *Providencia stuartii*, is synthesized as an inactive pro-protein with an N-terminal extension of eight amino acids (Stevenson *et al*., 2007). Activation of this TatA protein requires the regulated removal of the N-terminal extension by the membrane-bound rhomboid protease, AarA (Stevenson *et al*., 2007; Strisovsky *et al*., 2009). The active site of the rhomboid protease localizes close to the periplasmic side of the membrane, consistent with the N-out topology of TatA (Wang *et al*., 2006; Maegawa *et al*., 2007). *P. stuartii* is a close relative of *E. coli* and the *P. stuartii* TatA protein can replace the function of *E. coli* TatA and TatE in *E. coli* strains lacking these Tat components (Stevenson *et al*., 2007). GlpG is the *E. coli* homologue of the AarA rhomboid protease and has been shown to process *P. stuartii* TatA *in vitro* (Strisovsky *et al*., 2009).

In this study we have taken advantage of the cross-functionality of the *P. stuartii* and *E. coli* TatA proteins to address how the N-terminal extension of the *P. stuartii* TatA pro-protein impedes Tat function. Our results suggest that the extension inhibits the formation of large assemblies of TatA by preventing interaction between TatA and TatC.

## Results

### The TatA protein of *P. stuartii* requires N-terminal processing by GlpG to function in *E. coli*

**Fig. 1:**
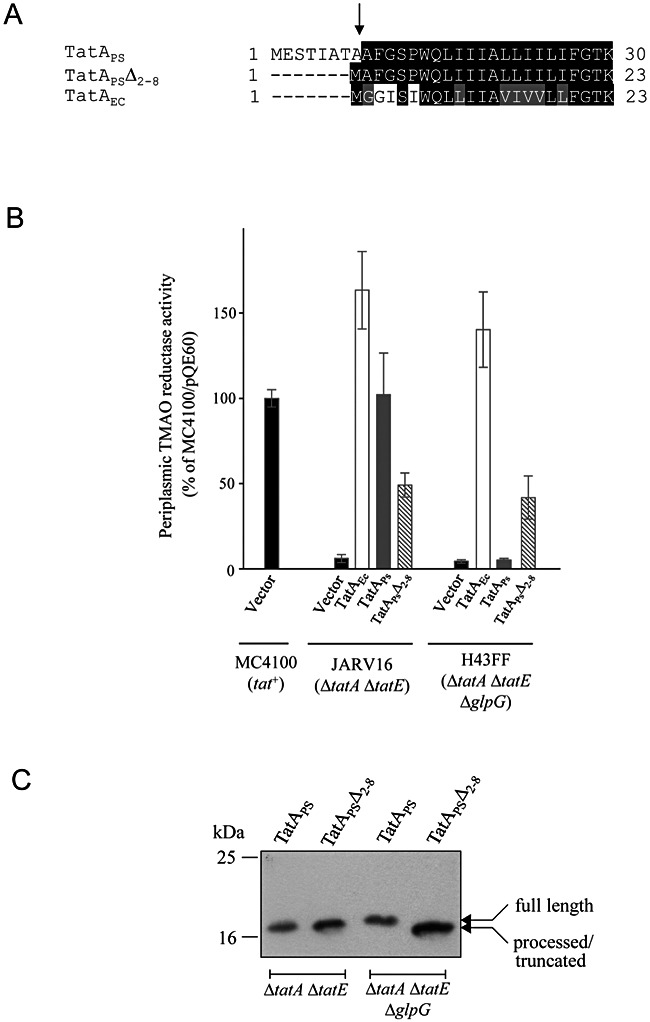
*P. stuartii* TatA is functional in *E. coli* but requires processing by GlpG for activity. A. Alignment of the N-termini of the *E. coli* and *P. stuartii* TatA proteins (TatA_Ec_ and TatA_Ps_ respectively), along with the truncated variant of *P. stuartii* TatA (TatA_Ps_Δ_2–8_) used in this study. Identical amino acids are shaded in black, conserved amino acids are shaded grey. The cleavage site for the rhomboid proteases AarA and GlpG is indicated with an arrow. B. *P. stuartii* TatA is active in *E. coli* in a *glpG*^+^ background. TMAO reductase activities were measured from the periplasmic fractions of the indicated strains carrying either pQE60 (labelled Vector), or pQE60 encoding C-terminally His-tagged variants of each of *E. coli* TatA (TatA_Ec_), *P. stuartii* TatA (TatA_Ps_) or a genetically truncated variant of *P. stuartii* TatA where codons 2–8 were lacking (TatA_Ps_Δ_2–8_). One hundred per cent activity is that determined from the periplasmic fraction of MC4100 harbouring pQE60 and corresponds to an activity of 4.7 µM benzyl viologen oxidized per min per mg protein. The error bars represent standard error of the mean (*n* = 5–8). C. *P. stuartii* TatA is processed *in vivo* by *E. coli* GlpG. Crude membrane fractions were prepared from *E. coli* strains JARV16-P (Δ*tatA*Δ*tatE*) and H43FF-P (Δ*tatA*Δ*tatE*Δ*glpG*) producing C-terminally His-tagged variants of the full-length or genetically truncated *P. stuartii* TatA proteins. Samples (1 µg membrane protein) were separated by SDS-PAGE (using a 10–20% Tris-tricine gradient gel) electroblotted and protein detected with a horseradish-peroxidase-conjugated penta-histidine antibody.

It was previously reported that the *P. stuartii* TatA protein (hereafter TatA_Ps_) can functionally replace *E. coli* TatA and TatE in phenotypic tests (Stevenson *et al*., 2007). However, the efficiency of this complementation, and whether the activity of TatA_Ps_ in *E. coli* was dependent upon the presence of the *E. coli* rhomboid protease GlpG, has not been assessed. To provide this information we deleted *glpG* in *E. coli* strain JARV16 (Δ*tatA*Δ*tatE*) and then used this genetic background to express plasmid-borne alleles coding for either C-terminally His-tagged *E. coli* TatA (TatA_Ec_), TatA_Ps_, or a truncated TatA_Ps_ variant (TatA_Ps_Δ_2–8_) lacking the rhomboid-sensitive N-terminal extension (deletion of amino acids two to eight; shown schematically in Fig. 1A). We cultured the strains anaerobically in the presence of trimethylamine-*N*-oxide (TMAO) and then assayed the activity of the Tat substrate TMAO reductase in the periplasmic fraction.

*E. coli* strain JARV16 expressing TatA_Ps_ had a periplasmic TMAO reductase activity that was indistinguishable from the Tat^+^*E. coli* parental strain (Fig. 1B) indicating that TatA_Ps_ can support a high level of Tat transport activity. In contrast, when *glpG* was also deleted from the Δ*tatA*Δ*tatE* mutant strain, expression of TatA_Ps_ no longer complemented the Δ*tatA*Δ*tatE* mutant phenotype. This was confirmed by the inability of this strain and plasmid combination to grow anaerobically on minimal media with TMAO as sole electron acceptor, or on media containing SDS [sensitivity to which arises due to the inability to export two Tat-dependent amidases involved in cell wall remodelling (Bernhardt and de Boer, 2003; Ize *et al*., 2003); data not shown]. As expected, plasmid-borne *tatA_Ec_* restored periplasmic export of TMAO reductase to the Δ*tatA*Δ*tatE* mutant strain, JARV16, regardless of whether GlpG was present or not, confirming that the activity of *E. coli* TatA does not depend upon processing by GlpG. The truncated TatA_Ps_Δ_2–8_ variant restored TMAO reductase export in *E. coli* strains lacking native TatA and TatE, and this complementation was independent of GlpG. However, it should be noted that TatAΔ_2–8_ supported a considerably lower periplasmic TMAO reductase activity than found for the wild-type *E. coli* strain. This suggests that the genetically truncated *P. stuartii* TatA variant is less active than the GlpG-processed protein. This difference in activity may be ascribed to the initiator methionine found only on the genetic truncation.

The results presented in Fig. 1B show that the Tat-dependent activity of TatA_Ps_ in *E. coli* strains depends on the presence of GlpG. To ascertain whether this was associated with processing of TatA_Ps_ to a shorter form, Western blot analysis of *P. stuartii* TatA was performed using the C-terminal His-tag as an epitope. These experiments were carried out using the same *E. coli*Δ*tatA*Δ*tatE* mutant strains described above but additionally containing the *pcnB1* allele to restrict plasmid copy number and avoid saturation of GlpG protease activity by overproduced TatA_Ps_. In the *glpG*^+^ background, JARV16-P, the TatA_Ps_ protein has the same mobility as the genetically truncated TatA_Ps_Δ_2–8_ variant (Fig. 1C). By contrast, in the *glpG* mutant strain, H43FF-P, the TatA_Ps_ protein migrated with a slightly lower mobility than the truncated form. These results indicate that all of the TatA_Ps_ is fully cleaved by GlpG, and that GlpG is the only protease in *E. coli* that can process the pro-protein. These findings are fully consistent with *in vitro* experiments showing that the transmembrane domain of *P. stuartii* TatA can be cleaved by purified GlpG (Stevenson *et al*., 2007; Strisovsky *et al*., 2009).

We next tested whether the *tatA_Ps_* gene showed genetic dominance, i.e. whether the inactive TatA_Ps_ protein that is produced in the absence of GlpG interferes with the function of wild-type *E. coli* TatA. To this end, plasmid-borne *tatA_Ps_* was expressed in both wild-type and Δ*glpG* strains (MC4100 and H1FF respectively). No reduction in periplasmic TMAO reductase activity was observed in either strain (Fig. S1). We therefore conclude that the pro-form of *P. stuartii* TatA protein does not interfere with the function of the native *E. coli* Tat machinery.

### The N-terminal extension of *P. stuartii* TatA does not prevent homo-oligomerization

One of the key features of TatA proteins is their ability to self-interact. Chemical cross-linking has been used to detect homo-oligomers of *E. coli* TatA, and of the thylakoid TatA orthologue Tha4, in membrane fractions (De Leeuw *et al*., 2001; Dabney-Smith *et al*., 2006; Greene *et al*., 2007). We therefore used chemical cross-linking to ascertain whether oligomerization of the *P. stuartii* TatA protein could be observed and whether this was affected by the presence of the N-terminal amino acid extension. Plasmid-encoded His-tagged TatA_Ps_ was produced in *E. coli*Δ*tatA*Δ*tatE* mutant strains that were either *glpG*^+^ (JARV16) or deleted for *glpG* (H43FF). Membrane fractions were then prepared from these strains, and from control strains producing His-tagged TatA_Ec_, and treated with the bifunctional chemical cross-linker disuccinimidyl suberate (DSS) which cross-links exposed amine residues.

**Fig. 2:**
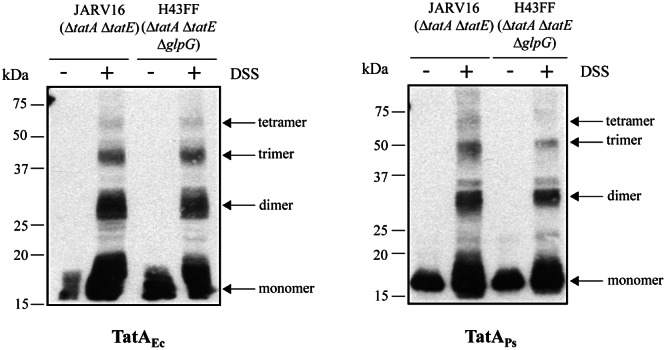
The N-terminal extension present on *P. stuartii* TatA does not prevent homo-oligomer formation. Crude membrane fractions were prepared from *E. coli* strains JARV16 (Δ*tatA*Δ*tatE*) and H43FF (Δ*tatA*Δ*tatE*Δ*glpG*) producing C-terminally His-tagged variants of either *E. coli* or *P. stuartii* TatA. Membrane fractions (30 µg protein) were treated with 2 mM DSS at pH 7.4 for 30 min. Samples (2 µg per lane) were subsequently separated by Tris-glycine SDS-PAGE (12.5% acrylamide), electroblotted and protein was detected with a horseradish-peroxidase-conjugated penta-histidine antibody. The positions of TatA multimers are indicated to the right-hand side and of molecular weight markers to the left-hand side of each blot.

Cross-linked species up to apparent homo-tetramers could be detected for both TatA_Ps_ and TatA_Ec_ (Fig. 2), consistent with an earlier study of DSS cross-linking of TatA_Ec_ (De Leeuw *et al*., 2001). Importantly, cross-linked oligomers of TatA_Ps_ were observed regardless of whether GlpG was present or absent, demonstrating that the N-terminal extension on the *P. stuartii* TatA protein does not prevent homo-oligomerization of the protein to at least the level of tetramer.

### The N-terminal extension of *P. stuartii* TatA inhibits the formation of large TatA complexes *in vivo*

**Fig. 3:**
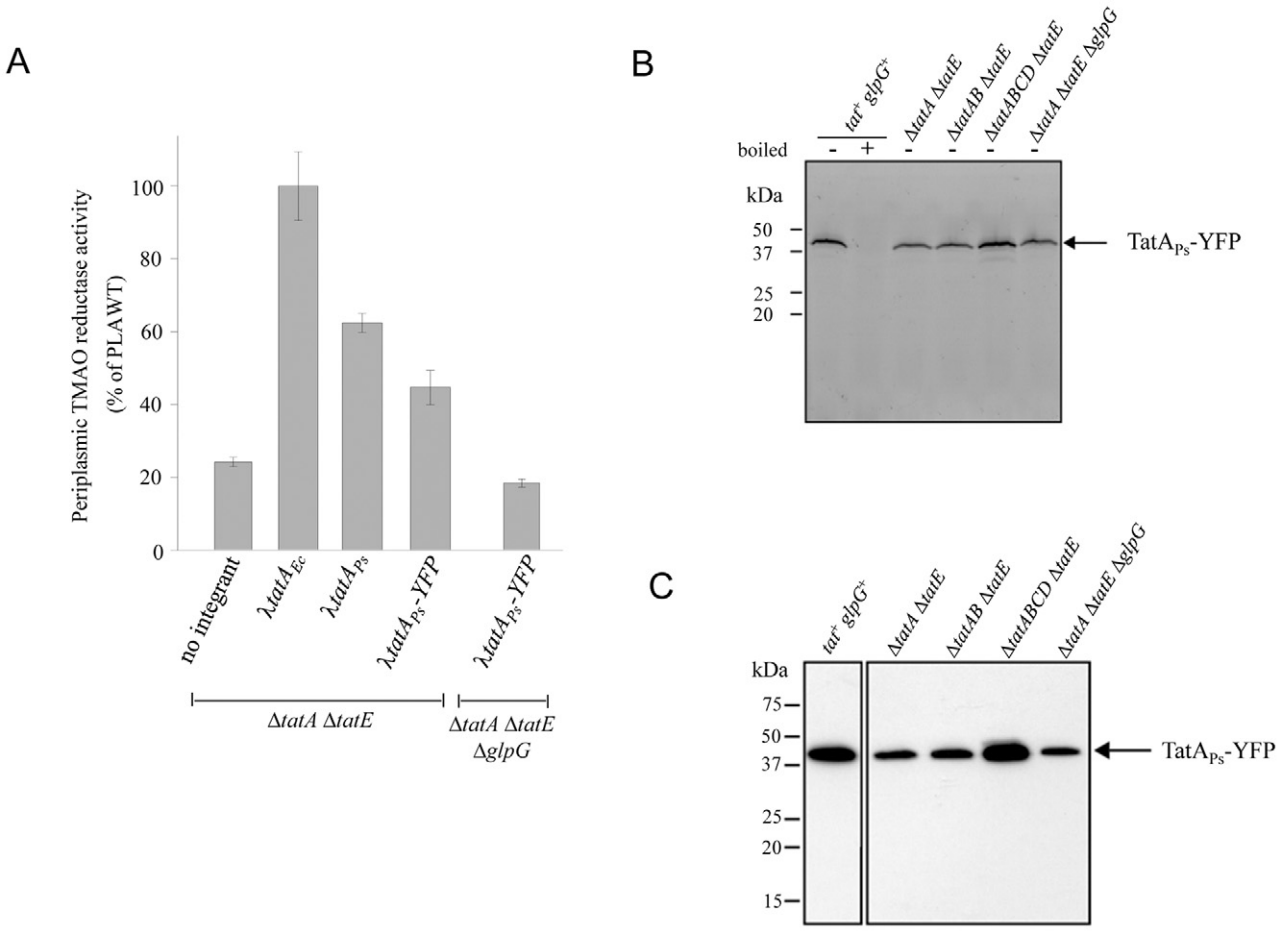
A *P. stuartii* TatA–YFP fusion protein is stably produced in *E. coli* and has TatA activity. A. *P. stuartii* TatA fused to YFP is active in *E. coli.* Periplasmic TMAO reductase activity was measured from strains deleted for the chromosomal *tatA* and *tatE* genes and expressing from the *attB* site: *E. coli tatA* (λ*tatA_Ec_*; strain PLAWT), *P. stuartii tatA* (λ*tatA_Ps_*; strain JARV16 λTatAPs) or a *P. stuartii tatA*–YFP fusion (λ*tatA_Ps_*–YFP; strain JARV16 λAPsALYFP). In addition activity was also measured from the strain producing the *P. stuartii tatA*–YFP fusion that was lacking *tatA*, *tatE* and *glpG* (strain H43FF λAPsALYFP). Activities shown are relative to that of the periplasmic fraction of strain PLAWT which corresponds to 3.3 µM benzyl viologen oxidized per min per mg protein. Error bars represent standard error of the mean (*n* = 3). B. The *P. stuartii* TatA–YFP fusion protein is fluorescent. Cell lysates (approximately 30 µg protein) of the strains MC4100 λAPsALYFP (*tat*^+^, *glpG*^+^), JARV16 λAPsALYFP (Δ*tatA*Δ*tatE*), BEAD λAPsALYFP (Δ*tatAB*Δ*tatE*), DADE λAPsALYFP (Δ*tatABCD*Δ*tatE*) and H43FF λAPsALYFP (Δ*tatABCD*Δ*tatE*Δ*glpG*) each producing the *P. stuartii* TatA–YFP fusion encoded at the *attB* site were separated by SDS-PAGE. The samples were not boiled prior to analysis with the exception of one of the MC4100 λAPsALYFP (*tat*^+^*glpG*^+^) samples, as indicated. Following SDS-PAGE the gel was excited with a laser at 473 nm and the fluorescent image was captured. C. The *P. stuartii* TatA–YFP fusion protein is stable. Un-boiled cell lysates of the indicated strains producing the *P. stuartii* TatA–YFP fusion protein were separated by SDS-PAGE, electroblotted and detected using an anti-GFP antibody. In (B) and (C) the molecular weight marker is shown to the left-hand side of the gel. An arrow at the right-hand side of the gel indicates the position of the *P. stuartii* TatA–YFP fusion.

It has previously been reported that a C-terminal YFP fusion of TatA_Ec_ forms distinct fluorescent foci containing tens of TatA_Ec_–YFP molecules in *E. coli* cells when both TatB and TatC are present (Leake *et al*., 2008). In the absence of TatB or TatC, these large TatA assemblies were not detected and instead TatA_Ec_–YFP was present in smaller clusters containing an average of four molecules. Since our data reported above supported the idea that the pro-form of TatA_Ps_ was still able to self-interact to form at least small oligomers, we next sought to test whether the formation of the large assemblies of TatA were prevented by the presence of the N-terminal amino acid extension. To achieve this, we constructed a TatA_Ps_–YFP fusion and expressed this under the control of the *E. coli tatA* promoter from the lambda phage attachment site of selected *E. coli* strains. Figure 3A shows the periplasmic TMAO reductase activity of *E. coli*Δ*tatA*Δ*tatE* strain JARV16, producing either TatA_Ec_, or TatA_Ps_, or TatA_Ps_–YFP from a single gene copy at the lambda attachment site. The strain producing TatA_Ps_–YFP has almost the same level of periplasmic TMAO reductase activity as the same strain producing the non-YFP-tagged *P. stuartii* TatA protein. If *glpG* was also absent from the Δ*tatA*Δ*tatE* strain, the TatA_Ps_–YFP protein was, as expected, not functional, with the periplasmic TMAO reductase activity measured from this strain being indistinguishable from that of the negative control. Taken together these results indicate that the YFP-tagged variant of TatA_Ps_ retains good Tat transport activity which is fully dependent upon N-terminal processing by GlpG.

Before the *in vivo* behaviour of the TatA_Ps_–YFP fusion protein was analysed, we first confirmed that the fusion protein was folded and stable. A single fluorescent species was detected in whole cell samples analysed by semi-native PAGE (Fig. 3B). Analysis of the same samples by denaturing SDS-PAGE and Western blotting with an anti-GFP antibody revealed a single immunoreactive band corresponding to the size of the fusion protein with no evidence of degradation to smaller forms (Fig. 3C). More fusion protein was present in the *tat*^+^ and Δ*tatABCD*Δ*tatE* backgrounds than in the other strains. The reason for this is unclear because all of the strains were constructed in an identical manner. Analysis of the subcellular localization of the fusion protein indicated that it was found exclusively in the membrane fraction in all strains (data not shown).

**Fig. 4:**
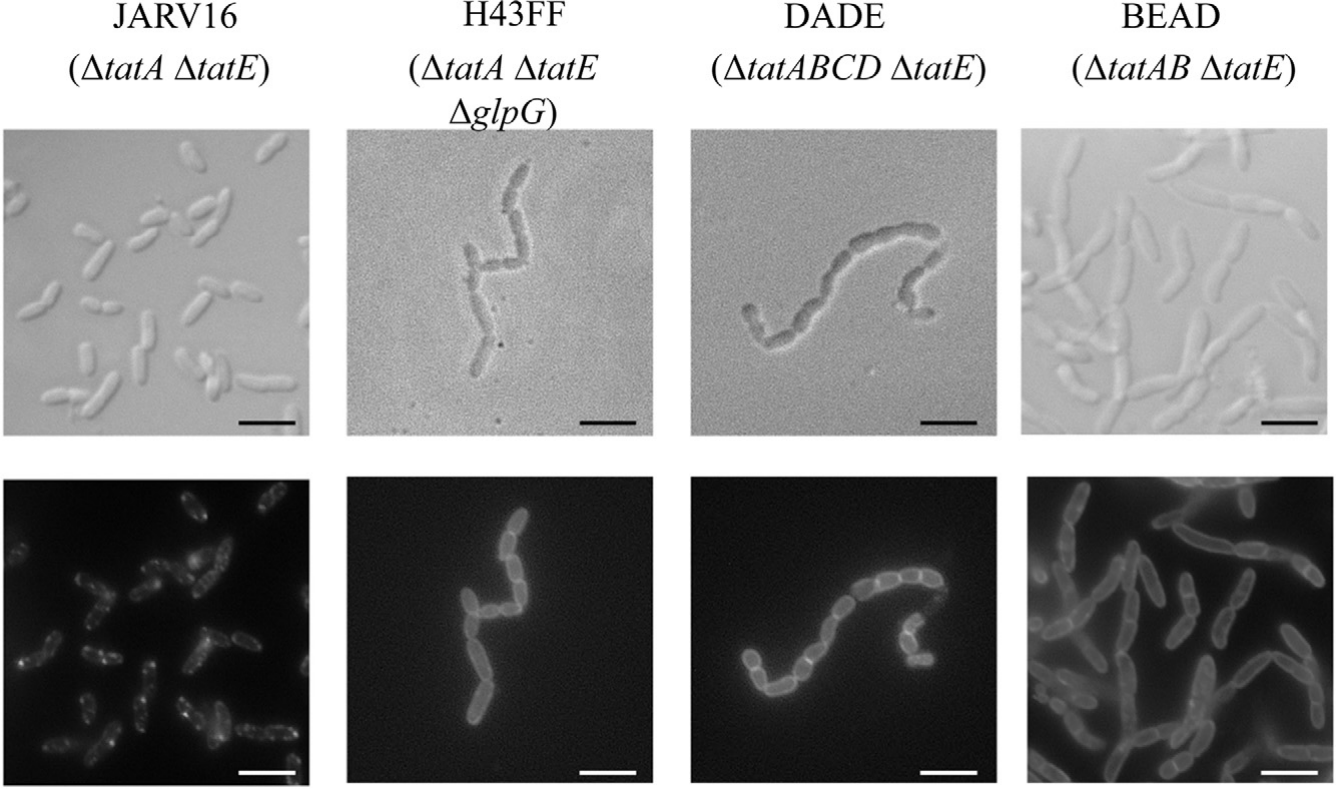
The N-terminal extension of *P. stuartii* TatA prevents the formation of large TatA assemblies *in vivo*. Fluorescence microscopy of strains JARV16 λAPsALYFP (Δ*tatA*Δ*tatE*), H43FF λAPsALYFP (Δ*tatABCD*Δ*tatE*Δ*glpG*), BEAD λAPsALYFP (Δ*tatAB*Δ*tatE*) and DADE λAPsALYFP (Δ*tatABCD*Δ*tatE*) producing the *P. stuartii* TatA–YFP fusion encoded at the *attB* site. The top images show cells in differential interference contrast (DIC) and bottom images show fluorescence of the *P. stuartii* TatA–YFP fusion protein. Scale bars correspond to 5 µm.

We next analysed the oligomerization behaviour of the TatA_Ps_–YFP fusion protein. When the fusion was produced in the *E. coli* JARV16 (Δ*tatA*Δ*tatE*) strain background, several large foci of fluorescence could be seen in each cell (Fig. 4). The TatA_Ps_–YFP protein, therefore, shows similar behaviour to the *E. coli* TatA–YFP fusion, which also forms large fluorescent foci when TatB and TatC are present (Leake *et al*., 2008). In the DADE (which lacks all *E. coli* Tat components) or BEAD (which produces only *E. coli* TatC) strain backgrounds, fluorescent foci of TatA_Ps_–YFP were not seen, and instead diffuse fluorescence could be seen all around the cell periphery, again as observed previously with *E. coli* TatA–YFP (Leake *et al*., 2008). Similarly a strain lacking all chromosomal *tat* genes and producing TatA_Ps_–YFP did not form fluorescent foci when TatB or TatC alone were produced from an inducible plasmid (Fig. S2). It should be noted that cells of these strains show a chaining morphology due to the failure to export Tat-dependent cell wall amidases (Bernhardt and de Boer, 2003; Ize *et al*., 2003). Importantly when the TatA_Ps_–YFP protein was observed in an *E. coli*Δ*tatA*Δ*tatE* background that was additionally deleted for *glpG*, the large fluorescent foci were also no longer seen, with only disperse fluorescence visible around the cell periphery. Taken together these results strongly suggest that the N-terminal extension on *P. stuartii* TatA inactivates Tat function by preventing the formation of large TatA assemblies *in vivo*.

As a control, we also examined the behaviour of the genetically truncated TatA_Ps_Δ_2–8_ variant fused to YFP. As expected, this YFP fusion protein formed foci in the Δ*tatA*Δ*tatE* background regardless of whether *glpG* was present or absent, and also corrected the chain-forming phenotype of the strain in a *glpG*-independent manner (Fig. S3).

### *E. coli* TatAC complexes can be isolated in the absence of TatB

In current models for Tat transport binding of a substrate to the TatBC complex in energized membranes primes TatBC to bind and polymerize TatA (e.g. Mori and Cline, 2002; Alami *et al*., 2003). Thus, one possible explanation for the failure of the pro-form of TatA_Ps_ to form large complexes may be that it is unable to interact with one or more components of the TatBC complex. Several lines of evidence indicate that TatA is likely to bind to the TatC component. However, to date a complex containing only *E. coli* TatA and TatC proteins has not been isolated.

**Fig. 5:**
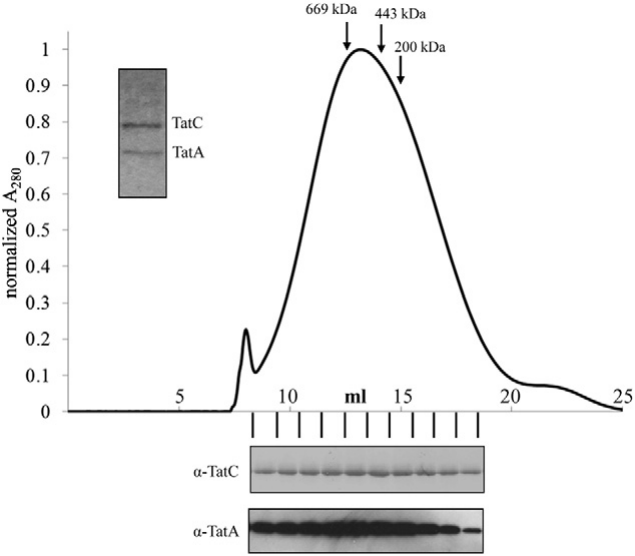
*E. coli* TatA co-purifies with TatC in the absence of TatB. TatA and TatC_his_ were co-produced in *E. coli* strain DADE (Δ*tatABCD*Δ*tatE*). Membrane proteins were solubilized in digitonin. His-tagged TatC and associated TatA was purified using nickel IMAC chromatography and visualized by Coomassie Blue staining after SDS-PAGE (inset). Purified TatAC_his_ complexes were subjected to gel filtration chromatography on a Superose 6 column and eluted as a broad peak (main image). Beta-amylase (200 kDa), apoferritin (443 kDa) and thyroglobulin (669 kDa) were used for molecular weight calibration (arrows). TatA and TatC were detected in the gel filtration fractions (indicated by lines) by Western blotting. All analysed fractions contained both TatA and TatC (bottom).

We investigated whether *E. coli* TatC and TatA were able to form complexes by co-producing TatA and hexahistidine-tagged TatC (TatC_his_) in the absence of other Tat components. Cell membranes were solubilized with 1% digitonin and the solubilized material was applied to a Ni^2+^-affinity column. TatC_his_ was eluted from the column using an imidazole gradient. Fractions containing TatC_his_ were pooled and subjected to gel filtration chromatography. Immunoblotting revealed TatA co-migrating with the affinity-purified TatC_his_ across a broad peak centred on an apparent molecular mass of approximately 500 kD (Fig. 5). These data indicate that *E. coli* TatA can interact directly with TatC in the absence of TatB. Instead of forming a single discrete complex, as seen for TatBC_his_ (Orriss *et al*., 2007), the TatAC_his_ complexes are heterogeneous. The yield of purified TatAC_his_ complexes was too low to allow further analysis. However, SDS-PAGE analysis of the concentrated affinity-purified fractions loaded on the gel filtration column revealed only two Coomassie Blue-staining bands corresponding to TatA and TatC_his_ (Fig. 5, inset). The relative staining intensities of the two bands suggest that TatA is not present at a significant molar excess over TatC_his_ in these complexes. Analysis of the unbound fraction from the Ni^2+^-affinity column showed that the vast majority of the TatA present in the soluble extract was not retained by the column and thus was not stably associated with TatC_his_ (data not shown).

### The N-terminal extension of *P. stuartii* TatA inhibits complex formation with TatC

To investigate whether the unprocessed N-terminal extension of *P. stuartii* TatA impaired interaction with TatC we developed a small-scale co-purification assay to allow parallel analysis of proteins expressed in multiple background strains. To this end, *E. coli* TatC_his_ was co-produced with either TatA_Ps_ or TatA_Ec_, membrane proteins were extracted with the detergent C_12_E_9_, and histidine-tagged TatC was purified from the solubilized material using Ni^2+^-affinity resin. The presence of TatA protein in the bound sample was then detected by Western blotting.

**Fig. 6:**
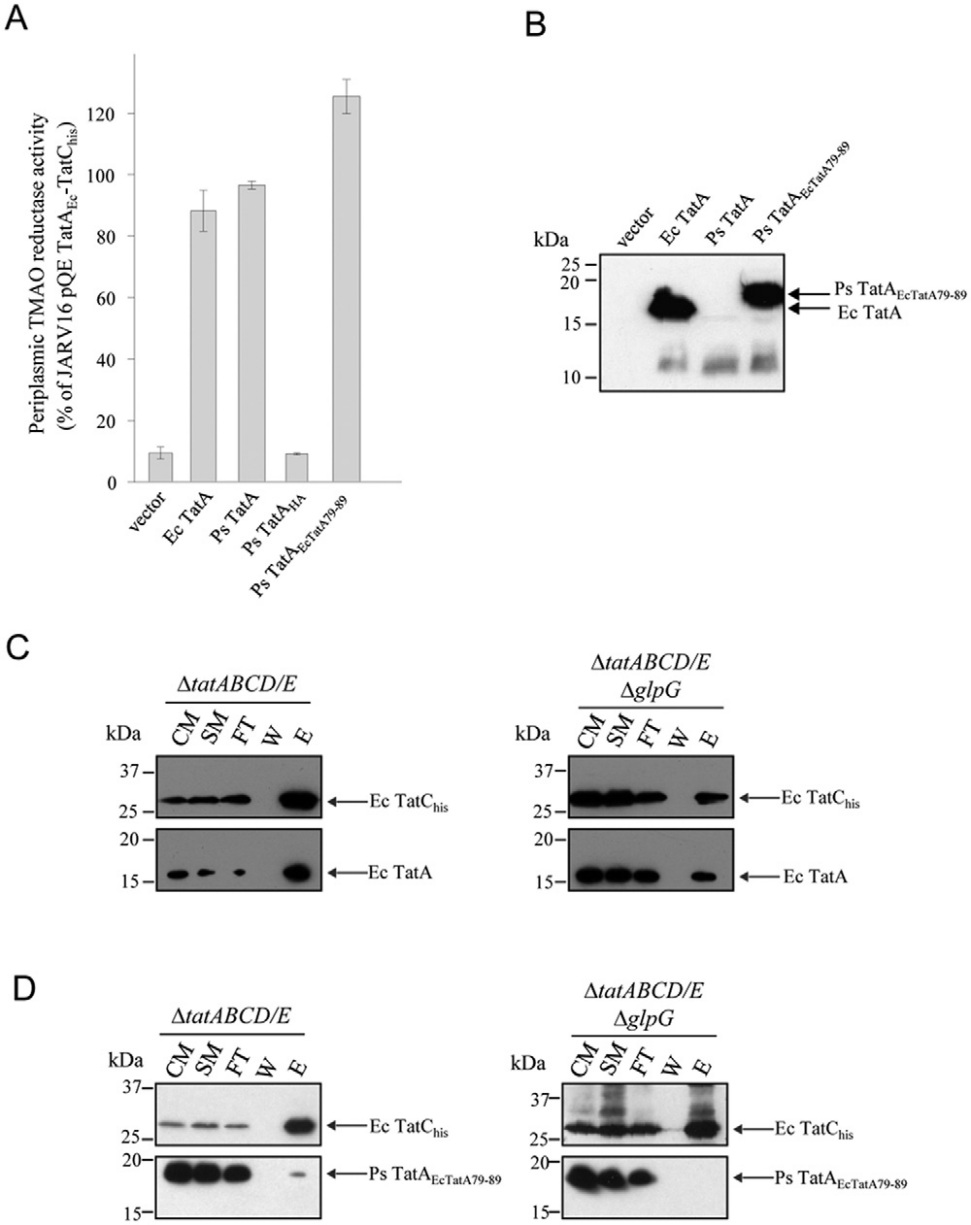
The N-terminal extension of *P. stuartii* TatA inhibits interaction with TatC. A. *P. stuartii* TatA with a C-terminal epitope from *E. coli* TatA is active in the *E. coli* Tat system. Periplasmic TMAO reductase activity was measured from *E. coli* strain JARV16-P (*tatA*Δ*tatE pcnB1*) harbouring plasmids producing His-tagged *E. coli* TatC_his_ in tandem with either *E. coli* TatA (TatA_Ec_), *P. stuartii* TatA (TatA_Ps_), *P. stuartii* TatA with a C-terminal haemagglutinin epitope (TatA_PsHA_) or *P. stuartii* TatA with a C-terminal epitope comprising the last 10 amino acids from *E. coli* TatA (TatA_PsEc_). The same strain harbouring plasmid pQE60 (vector) was used as a negative control. Activities shown are relative to that of the periplasmic fraction of JARV16/pQE TatA_Ec_-TatC_his_ and correspond to 5.7 µM benzyl viologen oxidized per min per mg protein. Error bars represent standard error of the mean (*n* = 3). B. *P. stuartii* TatA with a C-terminal epitope of *E. coli* TatA is detected by the anti-*E. coli* TatA antiserum. Cell lysates of JARV16-P containing pQE60 (vector) or producing His-tagged *E. coli* TatC in tandem with either *E. coli* TatA (TatA_Ec_), *P. stuartii* TatA (TatA_Ps_) or *P. stuartii* TatA with a C-terminal epitope from *E. coli* TatA (TatA_PsEc_) were separated by SDS-PAGE (15% acrylamide). Proteins were electroblotted and detected with an *E. coli* TatA antiserum. Arrows at the right-hand side of the blot indicate the positions of protein bands of TatA_Ec_ and TatA_PsEc_. Molecular weight markers are shown to the left-hand side of the blot. C and D. *E. coli* TatA or processed *P. stuartii* TatA co-purify with His-tagged *E. coli* TatC. Crude membrane fractions of the *E. coli* strain DADE (Δ*tatABCD*Δ*tatE*; left-hand panels in C and D) or H0FF (Δ*tatABCD*Δ*tatE*, Δ*glpG*; right-hand panels in C and D) both harbouring pREP4 (Zamenhof and Villarejo, 1972) and over-producing either *E. coli* TatA (TatA_Ec_) or epitope-tagged *P. stuartii* TatA (TatA_PsEc_) in tandem with hexa-histidine-tagged *E. coli* TatC (TatC_his_) were solubilized with detergent and the TatC_his_ protein purified using nickel-charged beads as described in *Experimental procedures*. Proteins that eluted from the beads were separated by SDS-PAGE (15% acrylamide), electroblotted and immunoreactive bands were detected with anti-tetra-histidine antibody (TatC blot, top) or anti-*E. coli* TatA antiserum (TatA blot, bottom). Samples are crude membrane fraction (CM), solubilized membrane fraction (SM), unbound fraction (U), wash (W), elution (E).

For these experiments it was necessary to provide *P. stuartii* TatA with a C-terminal epitope to allow detection, since our *E. coli* TatA antiserum does not detect TatA_Ps_. Initially we added a C-terminal haemagglutinin (HA) tag to give construct TatA_PsHA_. However, periplasmic TMAO reductase activity assays (Fig. 6A) show that this modification blocked Tat transport (and indeed the same tag also completely inactivated *E. coli* TatA; data not shown). Therefore, as an alternative approach, we tagged TatA_Ps_ with the last 10 C-terminal amino acids of *E. coli* TatA (forming TatA_PsEc_) since we had shown previously that this was the major epitope recognized by the *E. coli* TatA antiserum (Lee *et al*., 2002). The *E. coli*Δ*tatA*Δ*tatE* mutant strain, JARV16-P, producing TatA_PsEc_ had periplasmic TMAO reductase activity levels that were as high as those of cells expressing the corresponding constructs with *E. coli* TatA or untagged *P. stuartii* TatA (Fig. 6A). Additionally, it is clear from the Western blot shown in Fig. 6B that fusing the last 10 amino acids of *E. coli* TatA to the C-terminus of *P. stuartii* TatA results in strong recognition of the tagged *P. stuartii* TatA protein by the anti-*E. coli* TatA antiserum.

The co-purification assay was applied to *E. coli* TatA and TatC_his_ co-produced in a Δ*tatABCD*Δ*tatE* background using strain DADE. Blotting the eluted fraction with anti-TatA or anti-histag antibodies revealed that TatA_Ec_ and TatC_his_ were both present in the eluate confirming that the assay can detect the interaction between these two *E. coli* proteins (Fig. 6C). As expected, *E. coli* TatA co-purified with TatC_his_ regardless of whether the proteins were isolated from a *glpG*^+^ or a *glpG*^-^ strain (compare Fig. 6C left-hand and right-hand panels).

When the same experiments were repeated with epitope-tagged *P. stuartii* TatA protein, TatA_PsEc_ (Fig. 6D), the *P. stuartii* TatA protein co-purified with His-tagged *E. coli* TatC when the proteins were isolated from a *glpG*^+^ strain (left-hand panel). However, when the proteins were isolated from a *glpG* strain, none of the *P. stuartii* TatA protein was found in the eluted fraction, even though His-tagged TatC was clearly eluted from the resin (Fig. 6D, right-hand panel). To confirm that the binding of the *P. stuartii* TatA protein to the Ni^2+^-charged resin was due to interaction with TatC, and not to unspecific binding to the resin, control experiments were carried out where a non-tagged variant of *E. coli* TatC was used. In this case neither TatC nor the epitope-tagged *P. stuartii* TatA protein bound to the column, regardless of whether they were produced in a *glpG*^+^ or a *glpG* background (Fig. S4). Furthermore, we observed the same GlpG-dependent interaction of the (transport inactive) HA-tagged *P. stuartii* TatA protein with *E. coli* TatC_his_ (data not shown), indicating that the interaction is not dependent upon the nature of epitope tag. Taken together these results demonstrate that the N-terminal extension found on the *P. stuartii* TatA protein inhibits interaction with TatC.

As an additional control we assessed whether the genetically truncated variant of TatA_PsEc_, which lacked codons 2–8 could interact with *E. coli* TatC in a GlpG-independent manner. We found that this truncated TatA variant did not detectably co-purify with His-tagged *E. coli* TatC (Fig. S5). This is consistent with the observation that the genetically truncated TatA_Ps_ could not fully complement the Tat transport defect of the *E. coli tatA/E* mutant strain (Fig. 1) and suggests that the presence of the N-terminal methionine (which is lacking in the rhomboid-cleaved TatA_Ps_ variant) alters the stability of the TatAC complex so that it cannot survive detergent extraction or salt washing.

## Discussion

In this study we have used the inactive TatA protein from *P. stuartii*, which harbours a short N-terminal extension, as a tool to probe the Tat transport process. This protein is able to complement the Tat deficiency of an *E. coli*Δ*tatA*Δ*tatE* mutant strain provided that the *E. coli* rhomboid protease GlpG is present to cleave off the extension. This cross-complementation allowed us to take advantage of the range of *E. coli* genetic backgrounds and expression systems to probe the origin of the Tat defect associated with the unprocessed amino acid extension on TatA and to use this information to make inferences about the mechanism of Tat transport.

Previous studies have indicated that the *E. coli* TatA protein is able to self-interact to form complexes of varying size ranging from putative tetramers to much larger assemblies containing tens of subunits (De Leeuw *et al*., 2001; Porcelli *et al*., 2002; Gohlke *et al*., 2005; Oates *et al*., 2005; Leake *et al*., 2008). Similar behaviour has been inferred for the thylakoid TatA orthologue Tha4 (Dabney-Smith *et al*., 2006; Dabney-Smith and Cline, 2009). Our *in vitro* cross-linking experiments with the unprocessed form of TatA_Ps_ have shown that the N-terminal extension does not stop the TatA protein from forming small (tetrameric) homo-oligomers. However, *in vivo* imaging of a TatA_Ps_–YFP fusion revealed that the N-terminal extension prevented the formation of the large assemblies of TatA that were observed for the processed form of the protein and that have been previously reported for the *E. coli* TatA protein. We found that formation of large TatA_Ps_–YFP assemblies by the processed form of TatA requires the TatB and TatC proteins, again as previously observed for *E. coli* TatA (Leake *et al*., 2008). This raised the possibility that the unprocessed form of TatA_Ps_ was inactive because it was unable to interact with the TatBC complex.

The precise nature of the interaction of TatA with TatBC during Tat transport is not known. The purification of affinity-tagged TatA from cells overproducing all three *E. coli* proteins results in the co-purification of a low level of TatB, but no detectable TatC is co-purified (Sargent *et al*., 2001; de Leeuw *et al*., 2002). However, almost all of the TatB protein is found in a complex with TatC when affinity-tagged TatC is purified (e.g. Bolhuis *et al*., 2001; McDevitt *et al*., 2006). Very little TatA is associated in this TatBC complex, and it is not known with which of the proteins in the TatBC complex that TatA interacts. On the basis that variant *E. coli* TatA proteins have been isolated that are bifunctional and support a low level of Tat transport activity in the absence of TatB (Blaudeck *et al*., 2005), and that some Gram-positive Tat systems lack a TatB component (Jongbloed *et al*., 2006), we reasoned that TatA might interact directly with TatC during the Tat transport process rather than via TatB. Indeed, we were able to show that a stable interaction between *E. coli* TatC and TatA occurs in the absence of TatB. This observation is consistent with bimolecular fluorescence complementation data that indicate interaction between TatA and TatC in the absence of TatB (Kostecki *et al*., 2010) and site-specific cross-linking experiments that suggest that the transmembrane helix of TatA is close to TatC but not TatB (Frobel *et al*., 2011). Earlier analyses of *E. coli* strains expressing TatA and TatC in the absence of TatB failed to observe distinct TatC-containing complexes in solubilized membrane extracts (Barrett *et al*., 2007; Behrendt *et al*., 2007). This was ascribed to TatA interfering with the formation of the otherwise stable TatC complex or the formation of unstable TatAC complexes. Nevertheless, we have succeeded in isolating TatAC complexes. These complexes are apparently heterogeneous in composition and we speculate that this is why they were not observed in the earlier studies which were attempting to identify discrete TatC species.

Although the processed form of TatA_Ps_ was able to form a complex with TatC, similar to *E. coli* TatA, the unprocessed form of the protein could no longer be co-purified with TatC. This indicates that the N-terminal extension on TatA disrupts the interaction between TatA and TatC. Combining this idea with the observation that unprocessed TatA_Ps_ is also defective in the formation of large TatA complexes *in vivo* leads to the inference that substrate-induced polymerization of TatA is mediated by direct contact of TatC with TatA. Since strains co-producing either *E. coli* or *P. stuartii* TatA–YFP with TatC in the absence of TatB are unable to form large TatA complexes (Fig. 4; Leake *et al*., 2008) even though we are able to purify TatAC complexes containing small amounts of TatA (Figs 5 and 6C and D) we infer that without the assistance of TatB TatC can only bind a nucleus of TatA and is unable to drive full TatA polymerization. Our observations highlight the N-terminal region of TatA as playing a key role in the interaction with TatC. This is consistent with the findings of Blaudeck *et al*. (2005) who found that mutations falling within the first five N-terminal amino acids of TatA permitted the *E. coli* Tat pathway to function in the absence of TatB. It remains an open question whether the homologous TatA and TatB proteins have distinct binding sites on TatC or whether they alternately occupy the same site at different points during translocation.

## Experimental procedures

### Bacterial strains and growth conditions

Strains used in this study are listed in Tables 1 and S1. In general, *E. coli* strains were grown aerobically at 37°C in Luria–Bertani (LB) medium (Sambrook and Russell, 2001). Antibiotics were used at the following final concentrations; ampicillin – 100 µg ml^−1^, chloramphenicol – 25 µg ml^−1^, kanamycin – 50 µg ml^−1^, apramycin – 100 µg ml^−1^. Cultures (50 ml) for TMAO reductase activity measurements were grown anaerobically overnight at 37°C in LB containing 0.4% (w/v) TMAO and 0.5% (v/v) glycerol.

**Table 1:** Key strains and plasmids used in this study

Bacterial strain	Genotype	Reference
MC4100	F^-^, *[araD139]_B/r_*, Δ(*argF-lac*)*U169*, *λ*^-^, *e14-*, *flhD5301*, Δ(*fruK-yeiR*)*725*(*fruA25*), *relA1*, *rpsL150*(Str^R^), *rbsR22*, Δ(*fimB-fimE*)*632*(*::IS1*), *deoC1*	Casadaban (1976)
JARV16	MC4100, Δ*tatA*, Δ*tatE*	Sargent *et al*. (1999)
JARV16-P	MC4100, Δ*tatA*, Δ*tatE*, *pcnB1 zad-981*::Tn*10*d (Kan^R^)	Sargent *et al*. (1999)
BEAD	MC4100, Δ*tatAB,*Δ*tatE*	Lee *et al*. (2002)
DADE	MC4100, Δ*tatABCD,*Δ*tatE*	Wexler *et al*. (2000)
H1FF	MC4100, Δ*glpG*::*aac*(3)IV (Apra^R^)	This study
H43FF	MC4100, Δ*tatA*, Δ*tatE*, Δ*glpG*::*aac*(3)IV (Apra^R^)	This study
H43FF-P	MC4100, Δ*tatA*, Δ*tatE*, *pcnB1 zad-981*::Tn*10*d (Kan^R^), Δ*glpG*::*aac*(3)IV (Apra^R^)	This study
H0FF	MC4100, Δ*tatABCD,*Δ*tatE,*Δ*glpG*::*aac*(3)IV (Apra^R^)	This study
PLAWT	MC4100, Δ*tatA*, Δ*tatE*, *attB*::P*_tatA_*(*tatA^+^_E. coli_*)	Lee *et al*. (2002)
JARV16 λ*tatAPs*	MC4100, Δ*tatA*, Δ*tatE*, *attB*::P*_tatA_*(*tatA^+^_P. stuartii_*)	This study
H43FF λ*tatAPs*	MC4100, Δ*tatA*, Δ*tatE*, Δ*glpG*::*aac*(3)IV (Apra^R^), *attB*::P*_tatA_*(*tatA^+^_P. stuartii_*) (Kan^R^)	This study
MC4100 λAPsALYFP	MC4100, *attB*::P*_tatA_*(*tatA_P. stuartii_-tatA_E. coli_*^50–89^*–*YFP) (Kan^R^)	This study
JARV16 λAPsALYFP	MC4100, Δ*tatA*, Δ*tatE*, *attB*::P*_tatA_*(*tatA_P. stuartii_-tatA_E. coli_*^50–89^*–*YFP) (Kan^R^)	This study
BEAD λAPsALYFP	MC4100, Δ*tatAB,*Δ*tatE*, *attB*::P*_tatA_*(*tatA_P. stuartii_-tatA_E. coli_*^50–89^*–*YFP) (Kan^R^)	This study
DADE λAPsALYFP	MC4100, Δ*tatABCD,*Δ*tatE, attB*::P*_tatA_*(*tatA_P. stuartii_-tatA_E. coli_*^50–89^*–*YFP) (Kan^R^)	This study
H43FF λAPsALYFP	MC4100, Δ*tatA*, Δ*tatE*, Δ*glpG*::*aac*(3)IV (Apra^R^), *attB*::P*_tatA_*(*tatA_P. stuartii_-tatA_E. coli_*^50–89^*–*YFP) (Kan^R^)	This study

Plasmid	Characteristics	Reference
pQE60PsTatA	pQE60 encoding C-terminally His-tagged *P. stuartii* TatA	This study
pQE60PsTatAΔ2–8	pQE60 encoding C-terminally His-tagged *P. stuartii* TatA lacking codons 2–8 inclusive	This study
pFAT584	pQE60 encoding C-terminally His-tagged *E. coli* TatA	De Leeuw *et al*. (2001)
pQEA(ΔB)C	Overexpression of *E. coli tatA* and *tatC* under control of T5 promoter and *lac* operator; TatC produced without a His-tag	This study
pQEA(ΔB)Chis	Overexpression of *E. coli tatA* and *tatC* under control of T5 promoter and *lac* operator; codons for C-terminal His-tag on *tatC*	This study
pQEAPsHA(ΔB)Chis	Overexpression of *P. stuartii tatA* and *E. coli tatC* under control of T5 promoter and *lac* operator; codons for C-terminal His-tag on *tatC* and C-terminal haemagglutinin tag on *tatA*	This study
pQEAPsEc(ΔB)C	Overexpression of *P. stuartii tatA* and *E. coli tatC* under control of T5 promoter and *lac* operator; codons 79–89 of *E. coli* as C-terminal epitope on *P. stuartii tatA.* TatC produced without a His-tag	This study
pQEAPsEc(ΔB)Chis	Overexpression of *P. stuartii tatA* and *E. coli tatC* under control of T5 promoter and *lac* operator; codons for C-terminal His-tag on *tatC* and codons 79–89 of *E. coli* as C-terminal epitope on *P. stuartii tatA*	This study

Note that this is an abridged table and a full list of strains and plasmids can be found in Tables S1 and S2 respectively.

For chromosomal deletions of *glpG* the apramycin resistance cassette of plasmid pIJ790 (Gust *et al*., 2003) was amplified by PCR using primers glpGup and glpGdown (all oligonucleotides used in this study are listed in Table S3). This resulted in a PCR product where the apramycin cassette was flanked by 36 bp of sequence homologous to the up and downstream regions of *glpG,* including the start and stop codons respectively. Homologous recombination of the PCR product with the chromosomal *glpG*^+^ allele was carried out in *E. coli* strain BW25113 as described previously (Datsenko and Wanner, 2000) using the lambda Red recombinase expression plasmid pIJ773 (Gust *et al*., 2003). The disrupted *glpG* allele harbouring the apramycin resistance cassette was transferred from strain BW25113 Δ*glpG* to other *E. coli* strains by P1-mediated transduction using standard procedures (Miller, 1972).

### Plasmids

Plasmids used or constructed in this study are listed in Tables 1 and S2. Constructs pQE60PsTatA and pQE60PsTatAΔ2–8 harbour full-length *P. stuartii tatA* and a genetically truncated variant lacking codons 2–8, respectively, in pQE60. Full-length *P. stuartii tatA* was amplified by PCR using primers PStatANco and PstatABgl and the genetically truncated version using primers fwPs_NO_2–8 and rvPS_NO_2–8 with plasmid pBC.TatAPs (Stevenson *et al*., 2007) as a template. The PCR products were cloned into the multiple-cloning site of pQE60 using restriction enzymes NcoI and BglII.

The *E. coli tatA* promoter was fused to sequence of *P. stuartii tatA* as follows. The *tatA* promoter was amplified by PCR using primers UNIREP1 and coli/stuartiiblunt from MC4100 genomic DNA and digested with EcoRI. *P. stuartii tatA* was amplified using primers StuartiiTatAEcoRV and stuartirev2 with plasmid pBC.TatAPs (Stevenson *et al*., 2007) as template and digested with EcoRV and BamHI. The blunt end of the digested EcoRV site in the *P. stuartii tatA* PCR product was ligated directly to the undigested end of the PCR product with the *E. coli tatA* promoter and the resulting fragment was cloned into pLitmus28 (NEB) with EcoRI and BamHI, giving rise to construct pLitPstuaTatA. Plasmid pLitPstuaTatAstop was constructed by amplifying a PCR product with primers UNIREP1 and LitPstuatatAstop1 using plasmid pLitPstuatatA as template, and the product was cloned into pLitmus28 after digestion with EcoRI and BamHI. The *P. stuartii tatA* gene under control of the *E. coli tatA* promoter was excised from pLitPstuaTatAstop by digestion with EcoRI and BamHI and cloned into similarly digested pRS552 (Simons *et al*., 1987), resulting in construct pRSTatAPs. The *tatA* allele on this construct was subsequently integrated into the lambda attachment site on the chromosome of *E. coli* strains as described previously (Simons *et al*., 1987).

To construct *P. stuartii* TatA fused via an *E. coli* TatA linker region to YFP, first the *E. coli tatA* linker–*YFP* construct was assembled. Plasmid pTatA-NOSTOP2 contains the *E. coli tatA* gene with its native promoter in pBluescript (Leake *et al*., 2008). DNA covering *E. coli tatA* codons 50–89 fused to YFP was amplified using primers fwTATALINK and rvTATALINK using pTatA-NOSTOP2 as template. The PCR product was digested with EcoRI and BamHI and cloned into Bluescript, giving rise to plasmid pCTermTatAYFP. Subsequently the *P. stuartii tatA* gene under control of the *E. coli tat* promoter was amplified by PCR with primers UNIREP1 and TatAPsNsiI using pLitPstuaTatAstop as a template, and the PCR product was cloned into pCTermTatAYFP following digestion with EcoRI and NsiI, to give plasmid pAPsALYFP. The *tatA*–*YFP* gene fusion present in pAPsALYFP was subsequently excised by digestion with EcoRI and BamHI, and cloned into similarly digested pRS552, resulting in construct pRSAPsALYFP. Finally the *tatA*–*YFP* fusion was integrated into the lambda attachment site of *E. coli* strains according to the method of Simons *et al*. (1987).

To construct equivalent clones producing the genetically truncated variant lacking codons 2–8, a synthetic construct, pBSK-TatAPsD2–8, was purchased from Dundee Cell Products (Dundee, UK) comprising the promoter region, ribosome binding site and start codon of *E. coli tatA* and *P. stuartii* TatA starting from codon 8. The synthetic DNA fragment was cloned into pBluescript as an EcoRI–BamHI fragment and had an NsiI site introduced just prior to the *P. stuartii tatA* stop codon (sequence of this synthetic construct available upon request). A fragment covering the *E. coli tat* promoter-*P. stuartii tatA* truncated gene was excised by digestion with EcoRI and NsiI and cloned into similarly digested pAPsALYFP, thus replacing the full-length *P. stuartii tatA* gene on this construct with a fragment covering the genetic truncation, giving plasmid pAPsΔ2–8ALYFP. The truncated *tatA*–*YFP* gene fusion present in pAPsΔ2–8ALYFP was cloned into pRS552 as an EcoRI–BamHI fragment, giving construct pRSAPsΔ2–8ALYFP and integrated into the lambda attachment site of *E. coli* strains as before (Simons *et al*., 1987).

Plasmids for coexpression of *E. coli* or *P. stuartii tatA* along with *E. coli tatC* in vector pQE60 were constructed as follows. DNA covering *E. coli tatA* to the start of *tatB* was amplified using primers CANDIDA (Sargent *et al*., 1998) and TatBdelupXhoI2, with MC4100 chromosomal DNA as template, and the product was digested with EcoRI and XhoI. DNA covering the last few codons of *E. coli tatB* along with the entire *tatC* gene were amplified using TatBdeldownXho and either TatCBam (Lee *et al*., 2006; this primer includes the *tatC* stop codon) or TatCH2 (de Leeuw *et al*., 2002; this primer lacks the *tatC* stop codon) and MC4100 chromosomal DNA as template. These two DNA fragments were digested with XhoI and either BamHI (for the product generated with TatCBam) or BglII (for the product generated with TatCH2). The *tatB* deletion alleles were assembled by three-way ligation into pQE60 that had been digested with EcoRI and either BglII (for the fragment containing *tatC* lacking its stop codon) or BamHI (for *tatC* with its stop codon) to give plasmids pQEA(ΔB)Chis and pQEA(ΔB)C respectively. The *E. coli tatA* genes in plasmids pQEA(ΔB)C and pQEA(ΔB)Chis were replaced with variants of the *P. stuartii tatA* genes. For plasmid pQEAPs(ΔB)Chis, the wild-type *P. stuartii tatA* allele was amplified using primers TatA5 (Sargent *et al*., 1998) and TatAPsBEcXhoI and pLitPstuaTatAstop as template, the PCR product was digested with EcoRI and XhoI cloned first into pBluescript as an EcoRI–XhoI fragment and then excised as an EcoRI–EcoRV fragment and cloned into similarly digested pQEA(ΔB)Chis. Plasmid pQEAPsHA(ΔB)Chis was constructed by amplifying *P. stuartii tatA* using primers TatA5 (Sargent *et al*., 1998) and TatAPsHABEcXhoI (which includes DNA coding for a C-terminal haemagglutinin tag) with pLitPstuaTatAstop as a template and the PCR product was digested with EcoRI and XhoI and cloned first into pBluescript as an EcoRI–XhoI fragment and then excised as an EcoRI–EcoRV fragment and cloned into similarly digested pQEA(ΔB)Chis. Plasmids pQEAPsEc(ΔB)C and pQEAPsEc(ΔB)Chis contain the *P. stuartii tatA* gene fused to DNA coding for a C-terminal tag of the last 10 codons of *E. coli tatA*. The *P. stuartii tatA* gene was amplified using primers TatA5 (Sargent *et al*., 1998) and TatAPsEcBEcXhoI, with pLitPstuaTatAstop as a template. The resulting PCR product was digested with EcoRI and XhoI and cloned first into pBluescript as an EcoRI–XhoI fragment and then excised as an EcoRI–EcoRV fragment and cloned into each of pQEA(ΔB)C and pQEA(ΔB)Chis that had been similarly digested.

To construct a control plasmid producing the N-terminally truncated variant of *P. stuartii* TatA (with a C-terminal *E. coli* TatA epitope) along with histagged *E. coli* TatC in vector pQE60, DNA encoding the truncated *P. stuartii* TatA variant was amplified by PCR using primers CANDIDA and atAPsEcBEcXhoI with pBSK-TatAPsD2–8 as template. The DNA was first cloned into pBluescript as an EcoRI–XhoI fragment and subsequently excised as an EcoRI–EcoRV fragment and cloned into similarly digested pQEA(ΔB)Chis to give pQEΔ2–8ApsEc(ΔB)Chis.

### Protein methods

Periplasmic fractions were prepared using EDTA/lysozyme treatment and TMAO:benzyl viologen oxidoreductase activity in the periplasmic fraction was measured as described previously (Silvestro *et al*., 1988; Palmer *et al*., 2010a). SDS-PAGE and immunoblotting were carried out according to the methods of Laemmli (1970) and Towbin *et al*. (1979) respectively. Tris-Tricine gels were purchased from Bio-Rad. Antisera against *E. coli* TatC or *E. coli* TatA were used as described (Sargent *et al*., 2001; Alami *et al*., 2002) and immunoreactive bands were visualized with a chemiluminescent horseradish peroxidase substrate (Millipore). TatA–YFP fusion proteins were detected by immunoblotting using monoclonal Anti-Green Fluorescent Protein (GFP) antibody (Sigma-Aldrich, cat-Nr. G1546) and hexa-histidine-tagged TatA and TatC proteins were detected with anti-histidine antibodies (QIAGEN, Cat. No. 34670 and 34660). In-gel fluorescence of TatA–YFP fusion proteins after SDS-PAGE was detected by excitation with a laser at 473 nm in fluorescent image analyser FLA-5100 (Fujifilm) using filter LPB.

### Protein cross-linking

Crude membrane preparations and chemical protein cross-linking using DSS were performed as described by de Leeuw *et al*. (2001). Briefly, cell cultures (400 ml) were harvested and washed in 20 mM Na-MOPS (pH 7.2), 200 mM NaCl. The cells were disrupted with a French pressure cell at 8000 p.s.i. and after a short centrifugation step to remove unbroken cells, the crude membrane fraction was recovered by centrifugation at 200 000 *g* for 90 min. The pelleted membranes were resuspended in 1 ml of 20 mM Na-MOPS (pH 7.4), 200 mM NaCl. Protein (30 µg) in the crude membrane fraction was treated with 2 mM DSS (Sigma) in 20 mM K-HEPES (pH 7.4), 20 mM KCl, 250 mM sucrose, 1 mM EDTA at 25°C for 30 min and the reaction was stopped with 1 M Tris-HCl (pH 7.5) to a final concentration of 90 mM.

### Fluorescence microscopy

Cultures for fluorescence microscopy were grown aerobically at 37°C to an OD_600_ of 0.3. Cells were harvested by centrifugation, washed and resuspended in M9 minimal medium to one fifth of the original volume (Sambrook and Russell, 2001). Strain DADE-A λAPsALYFP expressing *tatB* alone, *tatC* alone or *tatB* and *tatC* together from plasmids pBAD33-tatB, pBAD24-tatC (both a kind gift from George Georgiou, University of Texas, Austin, USA) or pBAD-BC (Leake *et al*., 2008), respectively, was initially grown in LB medium supplemented with 0.2% glucose to repress transcription from the *araBAD* promoter (to prevent toxicity resulting from overexpression of *tatB* alone; Sargent *et al*., 1999). Cells were harvested, washed and resuspended as above in M9 minimal medium supplemented with 0.02% l-arabinose and incubated for 30 min to induce expression of plasmid encoded *tatB* and *tatC*. Cells were immobilized on coverslips using CellTak (BD Biosciences) and analysed by wide-field fluorescence microscopy (150× plan-apochromat objective, filter set 38He, Imager M1 microscope; Zeiss). Digital images were taken with a CCD camera (AxioCam MRm; Zeiss) and analysed using digital imaging software (Axio Vision LE 4.8; Zeiss).

### Purification of TatAC

A total of 4 × 750 ml of LB medium containing 0.4% (w/v) glycerol in baffled 2.5 l flasks was inoculated with 12.5 ml each of an overnight culture of *E. coli* DADE/pREP4/pQEA(ΔB)Chis and grown at 37°C to an OD_600_ of 0.7. IPTG was then added to a final concentration of 0.5 mM. After a further 3 h growth, the cells were harvested by centrifugation at 9000 *g* for 20 min. The cell pellet was resuspended in 20 ml of 20 mM MOPS/NaOH pH 8.0, 200 mM NaCl and broken by two passages through a French pressure cell at 14 000 p.s.i. Cell debris was pelleted by centrifugation at 12 000 *g* for 15 min before the supernatant was centrifuged at approximately 200 000 *g* for 1 h at 4°C to pellet the membrane fraction. The pellet was resuspended in 20 ml of 20 mM MOPS/NaOH, 200 mM NaCl before an equal volume of buffer containing 2% digitonin was added. After incubation at room temperature with gentle agitation for 3 h, the supernatant was cleared by centrifugation at 200 000 *g* for 1 h.

Imidazole was added to the supernatant to a final concentration of 20 mM and the resulting solution was applied to a 1 ml HisTrap (GE) column. The column was then washed with 10 column volumes of 20 mM MOPS pH 8, 200 mM NaCl, 20 mM imidazole, 0.15% digitonin. A linear gradient of 20–500 mM imidazole in 15 column volumes of the same buffer was then applied and protein-containing fractions were collected. After addition of 10 mM EDTA to scavenge nickel ions that may have leached from the resin, fractions containing TatAC complexes were pooled and concentrated to a volume of 0.5 ml using a centrifugal concentrator with a 100 kDa cut-off (Amicon). The concentrate was then applied to a Superose 6 10/300 (GE Healthcare) gel filtration column equilibrated with 20 mM MOPS pH 8, 200 mM NaCl, 0.15% digitonin. Fractions were analysed by Western blotting.

### Protein co-purification assay

*Escherichia coli* strains DADE/pREP4 or H0FF/pREP4 were used for co-purification assays of TatA and TatC proteins produced from plasmids pQEA(ΔB)Chis, pQEAPsEc(ΔB)C or pQEAPsEc(ΔB)Chis. Freshly transformed cells were grown in 500 ml of LB medium at 37°C with shaking to an OD_600_ of 0.6 and protein expression was induced with 2 mM IPTG at 25°C for 16 h. Cells were harvested by centrifugation, washed and resuspended in 25 ml of resuspension buffer (20 mM Na-HEPES (pH 7.2), 200 mM NaCl) supplemented with 1 mM PMSF (Sigma). Cells were disrupted with a French pressure cell (Thermo) at 8000 psi and the crude membrane fraction was pelleted by centrifugation at 200 000 *g* for 90 min at 4°C. The crude membrane pellet was resuspended in 1 ml of wash buffer (20 mM Na-HEPES (pH 7.2), 200 mM NaCl, 15 mM imidazole, 0.01% C_12_E_9_). Membrane proteins in the crude membrane fraction were solubilized at 4°C for 1 h with 1% C_12_E_9_ at a total protein concentration of 5 mg ml^−1^ and unsolubilized material was pelleted by centrifugation at 270 000 *g* for 30 min at 4°C. Nine hundred microlitres of solubilized membrane fraction was mixed with 100 µl of Profinity IMAC Ni-Charged resin (Bio-Rad) and gently pelleted by centrifugation at 400 *g* for 1 min at 4°C. The supernatant was retained as the unbound sample and the resin was washed four times with 1 ml wash buffer followed by gentle centrifugation as above. The supernatant after the last wash was retained and the resin was resuspended in 100 µl of elution buffer [20 mM Na-HEPES (pH 7.2), 200 mM NaCl, 300 mM imidazole, 0.01% C_12_E_9_]. The resin was pelleted by centrifugation at 400 *g* for 1 min at 4°C and the supernatant was kept as the eluted fraction. Subsequently, samples of the crude membrane fraction, the solubilized membrane fraction, the unbound fraction, the last wash fraction and the final eluted fraction were mixed 1:1 with 2× Laemmli sample buffer. Samples for Western blots against TatA were diluted 200-fold due to higher expression of *tatA* compared with *tatC* from the constructs. Finally, 5 µl of each sample was separated by SDS-PAGE and subsequently subjected to Western blotting using anti-TatA antiserum and anti-tetra-histidine tag antibodies, or anti-TatC antiserum in the case of untagged TatC.
