# Supplementary material for: Processing by rhomboid protease is required for Providencia stuartii TatA to interact with TatC and to form functional homo-oligomeric complexes
Source: Mol Microbiol. 2012 May 17;84(6):1108–23. doi: 10.1111/j.1365-2958.2012.08080.x (PMC3712462; doi:10.1111/j.1365-2958.2012.08080.x)
Supplement: Supplementary file 1 [file mmi0084-1108-SD1.pdf]

**Bacterial strain**	**Genotype**	**Reference**
MC4100	F^{-}, *[araD139]*$_{B/r}$, Δ(*argF-lac*)*U169*, *λ*$^{-}$, *e14-*, *flhD5301*, Δ(*fruK-yeiR*)*725*(*fruA25*), *relA1*, *rpsL150*(Str^{R}), *rbsR22*, Δ(*fimB-fimE*)*632*(*::IS1*), *deoC1*	Casadaban (1976)
JARV16	MC4100, Δ*tatA*, Δ*tatE*	Sargent *et al*. (1999)
JARV16-P	MC4100, Δ*tatA*, Δ*tatE*, *pcnB1 zad-981*::Tn*10*d (Kan^{R})	Sargent *et al*. (1999)
BEAD	MC4100, Δ*tatAB, ΔtatE*	Lee, *et al*. (2002)
DADE	MC4100, Δ*tatABCD, ΔtatE*	Wexler *et al*. (2000)
DADE-A	As DADE, ara^{+}	Ize *et al*., (2002)
BW25113	F^{-}, *rrnB3* Δ*lacZ4787 hsdR514* Δ*(araBAD)567* Δ*(rhaBAD)568 rph-1*	(Datsenko & Wanner, 2000)
BW25113 Δ*glpG*	BW25113, Δ*glpG*::*aac*(3)IV (Apra^{R})	this study
H1FF	MC4100, Δ*glpG*::*aac*(3)IV (Apra^{R})	this study
H43FF	MC4100, Δ*tatA*, Δ*tatE*, Δ*glpG*::*aac*(3)IV (Apra^{R})	this study
H43FF-P	MC4100, Δ*tatA*, Δ*tatE*, *pcnB1 zad-981*::Tn*10*d (Kan^{R}), Δ*glpG*::*aac*(3)IV (Apra^{R})	this study
H0FF	MC4100, Δ*tatABCD, ΔtatE,* Δ*glpG*::*aac*(3)IV (Apra^{R})	this study
PLAWT	MC4100, Δ*tatA*, Δ*tatE*, *attB*::P$_{tatA}$(*tatA*$^{+}_{E. coli}$)	Lee, *et al*. (2002)
JARV16 λ*tatAPs*	MC4100, Δ*tatA*, Δ*tatE*, *attB*::P$_{tatA}$(*tatA*$^{+}_{P. stuartii}$)	this study
H43FF λ*tatAPs*	MC4100, Δ*tatA*, Δ*tatE*, Δ*glpG*::*aac*(3)IV (Apra^{R}), *attB*::P$_{tatA}$(*tatA*$^{+}_{P. stuartii}$) (Kan^{R})	this study

Bacterial strain	Genotype	Reference
MC4100 λAPsALYFP	MC4100, *attB*::P_{tatA}(*tatA*$_{P.\ stuartii}$-*tatA*$_{E.\ coli}^{50\text{-}89}$-YFP) ($Kan^R$)	this study
JARV16 λAPsALYFP	MC4100, Δ*tatA*, Δ*tatE*, *attB*::P_{tatA}(*tatA*$_{P.\ stuartii}$-*tatA*$_{E.\ coli}^{50\text{-}89}$-YFP) ($Kan^R$)	this study
BEAD λAPsALYFP	MC4100, Δ*tatAB,* Δ*tatE*, *attB*::P_{tatA}(*tatA*$_{P.\ stuartii}$-*tatA*$_{E.\ coli}^{50\text{-}89}$-YFP) ($Kan^R$)	this study
DADE λAPsALYFP	MC4100, Δ*tatABCD,* Δ*tatE, attB*::P_{tatA}(*tatA*$_{P.\ stuartii}$-*tatA*$_{E.\ coli}^{50\text{-}89}$-YFP) ($Kan^R$)	this study
DADE-A λAPsALYFP	MC4100, Δ*tatABCD,* Δ*tatE, ara*$^+$ *attB*::P_{tatA}(*tatA*$_{P.\ stuartii}$-*tatA*$_{E.\ coli}^{50\text{-}89}$-YFP) ($Kan^R$)	this study
H43FF λAPsALYFP	MC4100, Δ*tatA*, Δ*tatE*, Δ*glpG*::*aac*(3)IV ($Apra^R$), *attB*::P_{tatA}(*tatA*$_{P.\ stuartii}$-*tatA*$_{E.\ coli}^{50\text{-}89}$-YFP) ($Kan^R$)	this study
JARV16 λ Δ2-8APsALYFP	MC4100, Δ*tatA*, Δ*tatE*, *attB*::P_{tatA}(*tatA*$_{P.\ stuartii\Delta 2\text{-}8}$-*tatA*$_{E.\ coli}^{50\text{-}89}$-YFP) ($Kan^R$)	this study
BEAD λ Δ2-8APsALYFP	MC4100, Δ*tatAB,* Δ*tatE*, *attB*::P_{tatA}(*tatA*$_{P.\ stuartii\Delta 2\text{-}8}$-*tatA*$_{E.\ coli}^{50\text{-}89}$-YFP) ($Kan^R$)	this study
DADE λ Δ2-8APsALYFP	MC4100, Δ*tatABCD,* Δ*tatE, attB*::P_{tatA}(*tatA*$_{P.\ stuartii\Delta 2\text{-}8}$-*tatA*$_{E.\ coli}^{50\text{-}89}$-YFP) ($Kan^R$)	this study
H43FF λ Δ2-8APsALYFP	MC4100, Δ*tatA*, Δ*tatE*, Δ*glpG*::*aac*(3)IV ($Apra^R$), *attB*::P_{tatA}(*tatA*$_{P.\ stuartii\Delta 2\text{-}8}$-*tatA*$_{E.\ coli}^{50\text{-}89}$-YFP) ($Kan^R$)	this study

Table S1. Complete list of strains used in this study

Plasmid	Characteristics	Reference
pIJ790	arabinose-inducible λ red recombinase on temperature sensitive replicon	Gust *et al*. (2003)
pIJ773	apramycin resistance gene flanked by FRT sites for disruption of genes by PCR targeting	Gust *et al*. (2003)
pCP20	temperature-sensitive replication and temperature-dependent induction of *S. cerevisiae* Flp recombinase.	Cherepanov & Wackernagel (1995)
pQE60	Cloning vector for producing histidine tagged proteins	Qiagen
pLitmus28	General cloning vector	New England Biolabs
pBluescript	General cloning vector	Stratagene
pREP4	*lacI,* kan^R	Zamenhof & Villarejo (1972)
pBC.TatAPs	700 bp fragment of *Providencia stuartii* (GenBank DQ989793) containing part of *ubiB*, full length *tatA* and part of *tatB* cloned into pBluescript SK as *Bam*HI/*Hinc*II fragment.	Stevenson *et al.* (2007)
pQE60PsTatA	pQE60 encoding C-terminally histagged *P. stuartii* TatA	this study
pQE60PsTatAΔ2-8	pQE60 encoding C-terminally histagged *P. stuartii* TatA lacking codons 2-8 inclusive.	this study
pFAT584	pQE60 encoding C-terminally histagged *E. coli* TatA	De Leeuw *et al.* (2001)
pLitPstuaTatA	pLitmus28 containing *P. stuartii tatA* without stop codon under control of the *E. coli tatA* promoter	this study
pLitPstuaTatAstop	pLitmus28 containing *P. stuartii tatA* without stop codon under control of the *E. coli tatA* promoter	this study

Plasmid	Characteristics	Reference
pRS552	*lacZ* operon fusion vector used for integration of genes as lambda pro-phage into the chromosomal *attB* site of *E. coli* strains	Simons *et al*. (1987)
pRSTatAPs	pRS552 containing *P. stuartii tatA* under control of the *E. coli tatA* promoter	this study
pBSK-TatAPsD2-8	pBluescript containing *P. stuartii tatA* deleted for codons 1-8 under control of the *E. coli tatA* promoter, ribosome binding site and start codon	this study
pCTermTatAYFP	*E. coli tatA* linker (codons 50-89) fused to YFP	this study
pTatA-NOSTOP2	*E. coli tatA* without stop codon under control of *E. coli tatA* promoter	Leake *et al.* (2008)
pAPsALYFP	*P. stuartii tatA* fused to YFP with C-terminal *E. coli tatA* linker (codons 50-89) under control of *E. coli tatA* promoter	this study
pRSAPsALYFP	*P. stuartii tatA*-YFP fusion from pAPsALYFP cloned into pRS552	this study
pAPsΔ2-8ALYFP	*P. stuartii tatA* deleted for codons 1-8 and under control of the *E. coli tatA* promoter, ribosome binding site and start codon fused to YFP with C-terminal *E. coli tatA* linker (codons 50-89).	this study
pRSAPsΔ2-8ALYFP	N-terminally truncated *P. stuartii tatA*-YFP fusion from pAPsΔ2-8ALYFP cloned into pRS552	this study
pQEA(ΔB)C	Over-expression of *E. coli tatA* and *tatC* under control of T5 promoter and *lac* operator; TatC produced without a His-tag.	this study
pQEA(ΔB)Chis	Over-expression of *E. coli tatA* and *tatC* under control of T5 promoter and *lac* operator; codons for C-terminal His-tag on *tatC*	this study

Plasmid	Characteristics	Reference
pQEAPs(ΔB)Chis	Over-expression of *P. stuartii tatA* and *E. coli tatC* under control of T5 promoter and *lac* operator; codons for C-terminal His-tag on *tatC*	this study
pQEAPsHA(ΔB)Chis	Over-expression of *P. stuartii tatA* and *E. coli tatC* under control of T5 promoter and *lac* operator; codons for C-terminal His-tag on *tatC* and C-terminal haemagglutinin tag on *tatA*	this study
pQEAPsEc(ΔB)C	Over-expression of *P. stuartii tatA* and *E. coli tatC* under control of T5 promoter and *lac* operator; codons 79-89 of *E. coli* as C-terminal epitope on *P. stuartii tatA.* TatC produced without a His-tag	this study
pQEAPsEc(ΔB)Chis	Over-expression of *P. stuartii tatA* and *E. coli tatC* under control of T5 promoter and *lac* operator; codons for C-terminal His-tag on *tatC* and codons 79-89 of *E. coli* as C-terminal epitope on *P. stuartii tatA*	this study
pQEΔ2-8ApsEc(ΔB)Chis	Over-expression of N-terminally truncated *P. stuartii tatA* and *E. coli tatC* under control of T5 promoter and *lac* operator; codons for C-terminal His-tag on *tatC* and codons 79-89 of *E. coli* as C-terminal epitope on *P. stuartii tatA*	this study

Table S2. Complete list of plasmids used in this study.

**Primer**	**Sequence (5' - 3')**
glpGup	ACTGTCCCCTTTTGTGTGGAATAAGCGACAGCAACGATGATTCCGGGGATCCG TCGACC
glpGdown	TGACGTTGTGTTTGTTTCATTTATAAATCCCTGGAATTATGTAGGCTGGAGCTG CTTC
PStatANco	GCGC**CCATGG**AATCAACTATTGCAACG
PstatABgl	GCGC**AGATCT**ACCCTGCTCTTTATTTTTGC
fwPs_NO_2-8	GCGC**CCATGG**CTTTTGGTAGCCCTTG
rvPS_NO_2-8	GCGC**AGATCT**ACCCTGCTCTTTATTTTTGCTCTC
UNIREP1	GCGC**GAATTC**TGTCGGTTGGCGCAAAACACGCTG
coli/stuartiiblunt	TCCATACATGTTCCTCTGTGGTAG
StuartiiTatAEcoRV	GCGC**GAATTCGATATC**AACTATTGCAACGGCCGC
stuartirev2	GCGC**GGATCCCGTACG**ACCCTGCTCTTTATTTTTGCTCTC
LitPstuatatAstop1	AAGCTTCGT**GGATCC**TTAACCCTGCTCTTTATTTTTGC
fwTATALINK	GCGC**GAATTCATGCAT**CAGGATAAAACCAGTCAGG
rvTATALINK	GCGC**GGATCC**TTACTTGTACAGCTC
TatAPsNsiI	GCGC**ATGCAT**ACCCTGCTCTTTATTTTTGCTCTCAACT
TatBdelupXhoI2	GCGC**CTCGAGGATATC**AAACACGGATTACACCTGC
TatBdeldownXho	GCGC**CTCGAG**CCTTCGTCGAGTGATAAACCGTAA
TatAPsBEcXhoI	GCGC**CTCGAGGATATC**AAACACGGATTAACCCTGCTCTTTATTTTTG
TatAPsEcBEcXhoI	GCGC**CTCGAGGATATC**AAACACGGATTACACCTGCTCTTTATCGTGGCGCTTC GCGTCACCCTGCTCTTTATTTTTGC
TatAPsHABEcXhoI	GCGC**CTCGAGGATATC**AAACACGGATTACGCATAGTCCGGCACATCGTACGG ATAACCCTGCTCTTTATTTTTGC

Table S3. Oligonucleotides used in this study

Figure S1. ***P. stuartii*** **TatA does not have a dominant negative effect on the activity of the native** ***E. coli*** **Tat system.** TMAO reductase activities were measured from the periplasmic fractions of the indicated strains carrying either pQE60 (labelled Vector), or pQE60 encoding C-terminally his-tagged variants of each of *E. coli* TatA ($TatA_{Ec}$), *P. stuartii* TatA ($TatA_{Ps}$) or a genetically truncated variant of *P. stuartii* TatA where codons 2-8 were lacking ($TatA_{Ps}\Delta_{2\text{-}8}$). 100% activity is that determined for MC4100 harbouring pQE60 and corresponds to an activity of 4.7 μM benzyl viologen oxidised per min per mg protein. The error bars represent standard error of the mean (*n* = 5).

Figure S2. TatB or TatC alone do not promote the formation of large TatA assemblies ***in vivo*****.** Fluorescence microscopy of strain DADE-A λAPsALYFP (Δ*tatABCD* Δ*tatE,* ara^+) producing the *P. stuartii* TatA-YFP fusion encoded at the *attB* site was transformed with a plasmid harbouring an L-arabinose inducible promoter encoding *tatB* alone (ptatB), *tatC* alone (ptatC) or *tatB* and *tatC* together (ptatBC). Cells were grown aerobically in LB medium supplemented with 0.2% glucose at 37°C to an OD_{600} of 0.3. Cells were harvested by centrifugation, washed and resuspended in one fifth of the original volume of M9 minimal medium supplemented with 0.02% L-arabinose and incubated for 30 minutes before microscopy analysis. The top images show cells in differential interference contrast (DIC) and bottom images show fluorescence of the *P. stuartii* TatA-YFP fusion protein. Scale bars correspond to 5 μm. Note that cells harbouring plasmid-encoded *tatBC* still show some cell chaining. This is because the relatively short time of induction of *tatBC* expression is insufficient to fully restore the wild type phenotype.

Figure S3. A genetically truncated variant of ***P. stuartii*** **TatA forms large TatA assemblies** ***in vivo*** **in a GlpG independent manner.** Fluorescence microscopy of strains JARV16 λ Δ2-8APsALYFP (Δ*tatA* Δ*tatE*), H43FF Δ2-8APsALYFP (Δ*tatABCD* Δ*tatE* Δ*glpG*), BEAD Δ2-8APsALYFP (Δ*tatAB* Δ*tatE*) and DADE Δ2-8APsALYFP (Δ*tatABCD* Δ*tatE*) producing the N-terminally truncated *P. stuartii* TatA-YFP fusion encoded at the *attB* site. The top images show cells

in differential interference contrast (DIC) and bottom images show fluorescence of the *P. stuartii* TatA-YFP fusion protein. Scale bars correspond to 5 μm.

Figure S4. Wild Type *E. coli* TatC and epitope-tagged *P. stuartii* TatA tagged do not bind to Ni^{2+}-charged IMAC resin. Crude membrane fractions of the *E. coli* strain DADE (Δ*tatABCD* Δ*tatE*; left hand panels) or H0FF (Δ*tatABCD* Δ*tatE*, Δ*glpG*; right hand panels) both harbouring pREP4 and over-producing epitope-tagged *P. stuartii* TatA ($TatA_{PsEc}$) in tandem with wild type *E. coli* TatC were solubilised with detergent and incubated with nickel-charged beads as described in Experimental Procedures. Protein samples were separated by SDS-PAGE (15% acrylamide), electroblotted and immunoreactive bands were detected with anti-*E. coli* TatC (TatC blot, top) or anti-*E. coli* TatA antiserum (TatA blot,bottom). Samples are crude membrane fraction (CM), solubilised membrane fraction (SM), unbound fraction (U), wash (W), elution (E).

Figure S5. The genetically truncated variant of *P. stuartii* TatA does not co-purify with TatC. Crude membrane fractions of the *E. coli* strain DADE (Δ*tatABCD* Δ*tatE*; left hand panels) or H0FF (Δ*tatABCD* Δ*tatE*, Δ*glpG*; right hand panels) both harbouring pREP4 and over-producing epitope-tagged and truncated *P. stuartii* TatA (Δ2-8$TatA_{PsEc}$) in tandem with hexa-histidine-tagged *E. coli* TatC ($TatC_{his}$) were solubilised with detergent and incubated with nickel-charged beads as described in Experimental Procedures. Protein samples were separated by SDS-PAGE (15% acrylamide), electroblotted and immunoreactive bands were detected with anti-tetra-histidine antibody (TatC blot, top) or anti-*E. coli* TatA antiserum (TatA blot, bottom). Samples are crude membrane fraction (CM), solubilised membrane fraction (SM), unbound fraction (U), wash (W), elution (E).

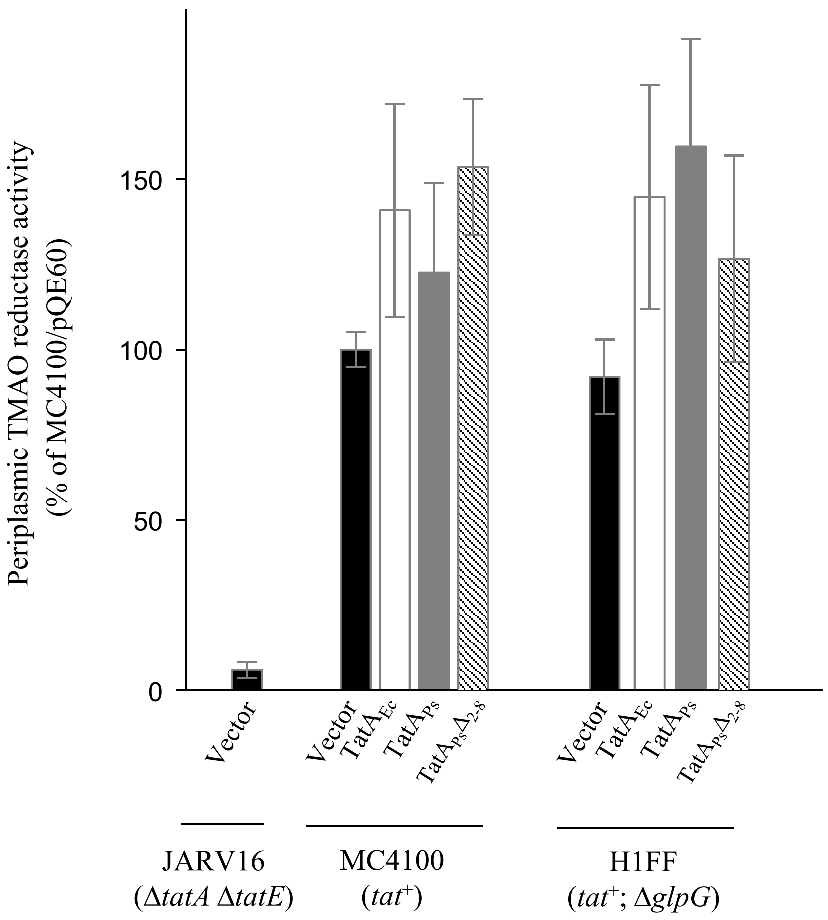

Fritsch *et al*. Fig S1

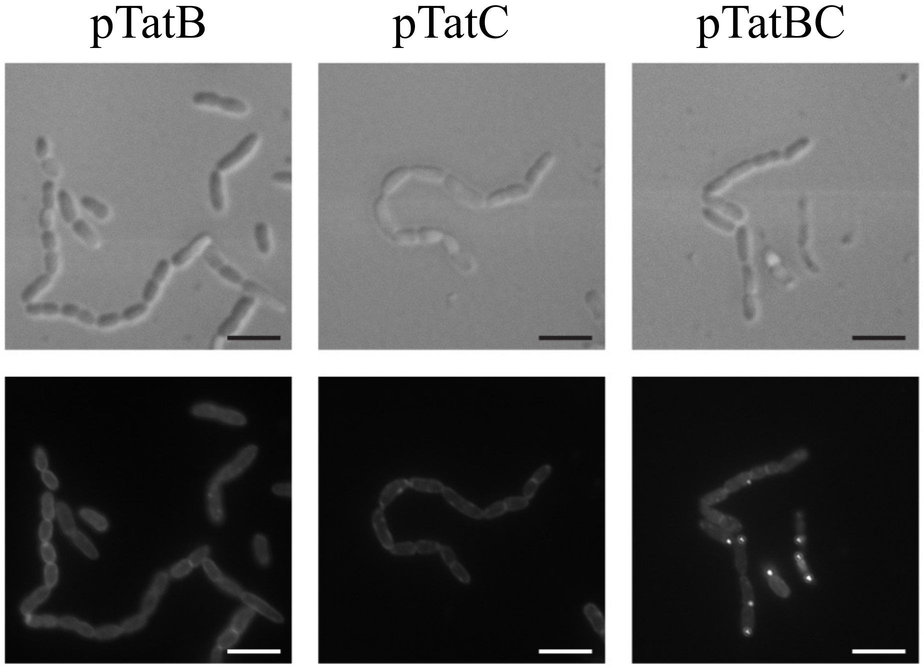

Fritsch *et al*. Fig S2

JARV16 (Δ*tatA* Δ*tatE*) | H43FF (Δ*tatA* Δ*tatE* Δ*glpG*) | DADE (Δ*tatABCD* Δ*tatE*) | BEAD (Δ*tatAB* Δ*tatE*)

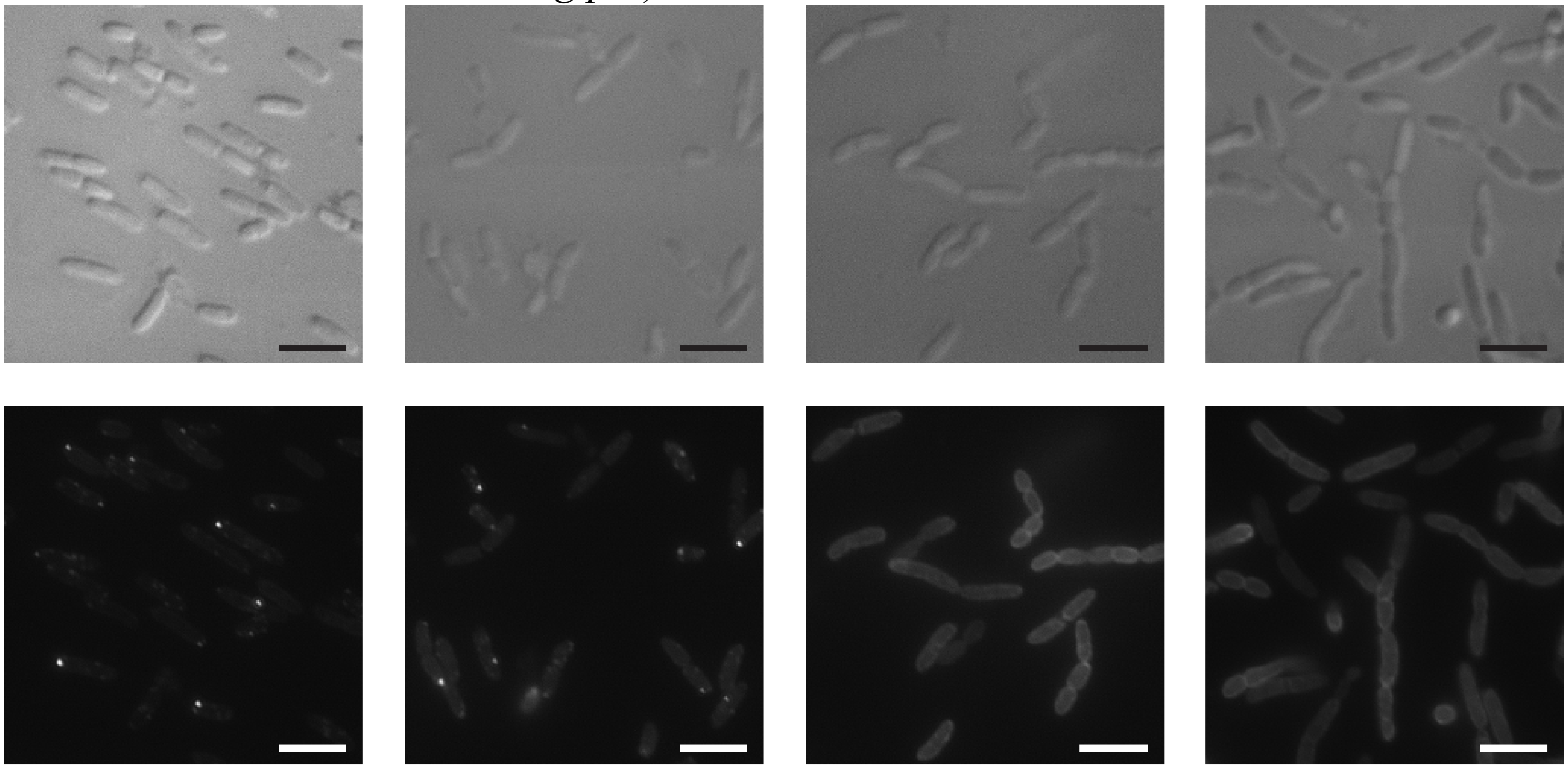

Fritsch *et al*. Fig S3

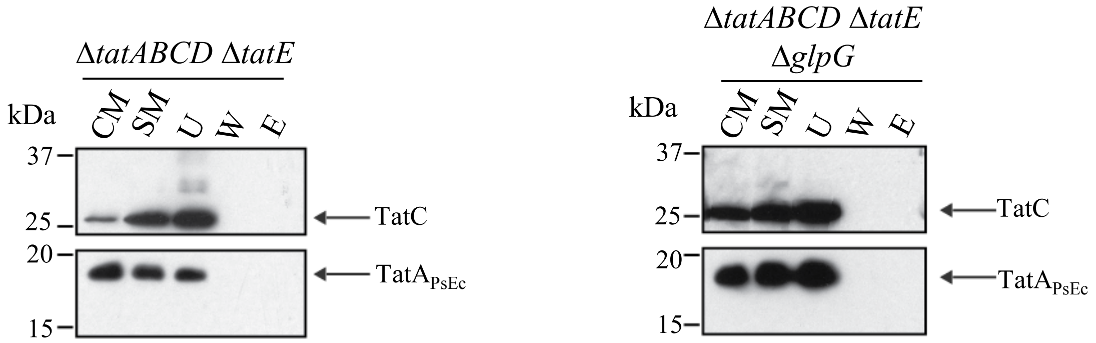

Fritsch *et al*. Fig S4

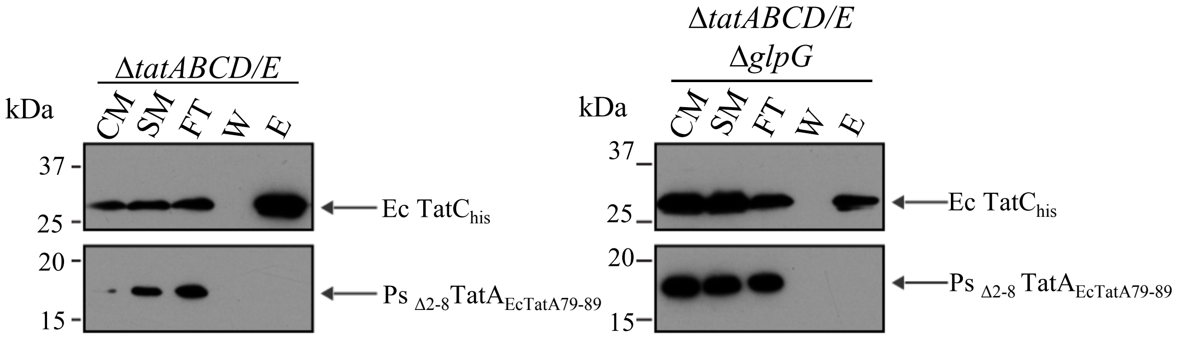

Fritsch *et al*. Fig S5
